# Prevalence and trends of coronary artery disease risk factors and their effect on age of diagnosis in patients with established coronary artery disease: Tehran Heart Center (2005–2015)

**DOI:** 10.1186/s12872-021-02293-y

**Published:** 2021-10-04

**Authors:** Kaveh Hosseini, Seyedeh Hamideh Mortazavi, Saeed Sadeghian, Aryan Ayati, Mahdi Nalini, Arya Aminorroaya, Hamed Tavolinejad, Mojtaba Salarifar, Hamidreza Pourhosseini, Afsaneh Aein, Arash Jalali, Ali Bozorgi, Mehdi Mehrani, Farin Kamangar

**Affiliations:** 1 Tehran Heart Center Research Institute, Tehran Heart Center, Tehran University of Medical Sciences, Tehran, Iran; 2 Cardiovascular Research Center, Kermanshah University of Medical Sciences, Kermanshah, Iran; 3 Non-Communicable Diseases Research Center, Endocrinology and Metabolism Population Sciences Institute, Tehran University of Medical Sciences, Tehran, Iran; 4 Department of Biology, School of Computer, Mathematical, and Natural Sciences, Morgan State University, Baltimore, MD USA

**Keywords:** Coronary artery disease, Cardiovascular risk factors, Prevention

## Abstract

**Background:**

Coronary artery disease (CAD) is a universal public health challenge, more prominently so in the low- and middle-income countries. In this study, we aimed to determine prevalence and trends of CAD risk factors in patients with documented CAD and to determine their effects on the age of CAD diagnosis.

**Materials and methods:**

We conducted a registry-based, serial cross-sectional study using the coronary angiography data bank of the Tehran Heart Center. Adult patients who had obstructive (> 50% stenosis) CAD were included in the study. The prevalence and 11-year trends of conventional CAD risk factors were analyzed by sex and age, and their adjusted effects on the age of CAD diagnosis were calculated.

**Results:**

From January 2005 to December 2015, data for 90,094 patients were included in this analysis. A total of 61,684 (68.5%) were men and 28,410 (31.5%) were women. Men were younger at diagnosis than women, with a mean age of 60.1 in men and 63.2 in women (*p* < 0.001), and had fewer risk factors at the time of diagnosis. Mean age at diagnosis had an overall increasing trend during the study period. Increasing trend was seen in body-mass index, hypertension prevalence, diabetes mellitus. All lipid profile components (total cholesterol, low-density lipoprotein cholesterol, triglycerides, and high-density lipoprotein cholesterol) decreased over time. Of particular interest, opium consumption was associated with 2.2 year earlier age of CAD diagnosis.

**Conclusion:**

The major results of this study (lower age of CAD diagnosis in men, lower age of diagnosis associated with most risk factors, and lower prevalence of serum lipids over time) were expected. A prominent finding of this study is confirming opium use was associated with a much younger age of CAD onset, even after adjusting for all other risk factors. In addition to recommendations for control of the traditional risk factors, spreading information about the potential adverse effect of opium use, which has only recently been associated with higher risk of CAD, may be necessary.

## Introduction

Obstructive coronary artery disease (CAD) is a leading cause of death worldwide, both in high-income and low- to middle-income countries (LMIC), including many countries in the Middle East [1, 2]. Over the past three decades, age-standardized mortality and years lived with disability (YLD) rates of CAD in the Middle East/North African region decreased by 35% and 9%, respectively [3], however, this region still has one of the highest burdens of cardiovascular diseases in the world [4, 5].

According to the Institute for Health Metrics and Evaluation, in 2019 ischemic heart disease was the leading cause of total and premature death in Iran, a country of nearly 85 million people in the Middle East [6]. It is estimated that from 2005 to 2025, DALYs attributable to cardiovascular diseases, including CAD, in adults aged ≥ 30 years, will increase by more than two-fold in Iran (from 847,309 DALYs in 2005 to 1,728,836 DALYs in 2025) [7, 8]. Therefore, careful investigations of the CAD risk factors and approaches to implement primary and secondary prevention protocols are necessary.

Hospital-based serial cross-sectional studies, despite their limitations and generalizability issues, provide unique insights about the trends of risk factors in patients with established CAD. They are also informative about the age of the onset of the disease in patients with CAD, and hence can determine whether obstructive CAD is occurring in younger ages or older ages over time. Such trends will provide important information about the success of prevention measures, and are important not only to policy makers but also to specialty physicians taking care of CAD patients.

In this study, we aimed to investigate the prevalence and 11-year trends of CAD risk factors among over 90,000 CAD patients seen at the Tehran Heart Center (THC), a tertiary referral center, for coronary angiography. We also investigated the prevalence of these risk factors between men and women, and studied their impacts on the age of diagnosis of CAD.

## Materials and methods

### Study setting and data source

THC is a major tertiary referral center in Tehran, Iran, dedicated to the treatment of heart diseases. Since 2001, on average each year approximately 76,000 patients are seen in outpatient clinics; nearly 47,000 patients are visited in the emergency rooms; 16,000 patients are hospitalized; over 3200 patients undergo open-heart surgeries; and 9000 coronary angiographies and over 2000 angioplasties are done at this center annually [9].

A coronary angiography databank was established in January 2005, which collects data from all patients who undergo angiography at THC. Demographic, clinical, and laboratory data are collected during the admission to catheterization laboratory and are recorded in an electronic registry. An informed consent is completed by patients before their data is included in the databank.

### Design and participants

In this registry-based serial cross-sectional study, baseline data from all patients in the THC coronary angiography databank enrolled from 2005 through 2015 were reviewed. Patients with obstructive CAD—defined by an angiography report of more than 50% stenosis in at least one vessel—were included in this study. The conventional CAD risk factors including hypertension, dyslipidemia, diabetes mellitus, cigarette smoking, body-mass index (BMI), low-density lipoprotein cholesterol (LDL-C), high-density lipoprotein cholesterol (HDL-C), triglycerides, total cholesterol, and fasting blood sugar (FBS) were analyzed according to sex and age, and the 10-year trends were assessed.

### Definitions of variables

*Hypertension* was defined, using two office measurements, as a mean systolic blood pressure ≥ 140 mmHg or a mean diastolic blood pressure of ≥ 90 mmHg or taking blood pressure medications. Blood pressure was measured using a manual mercury sphygmomanometer in all cases. Trained nurses measured blood pressure in both arms in a sitting position after resting for 5 min. The higher blood pressure was registered in a pre-specified data sheet. This protocol was repeated after 3 min. In case of difference (more than 10 mmHg in systolic blood pressure or 5 mmHg in diastolic blood pressure), the measurement was repeated for a third time and the two measurements closer together were used. Mean systolic and diastolic blood pressure of the two measurements was calculated. *Diabetes mellitus* was defined by measurements of fasting plasma glucose ≥ 126 mg/dL, glycated hemoglobin A1C (HbA1C) ≥ 6.5% in the presence of confirmatory testing, or taking anti-diabetic medications. *Dyslipidemia* was defined as total serum cholesterol ≥ 200 mg/dL, HDL-C < 40 mg/dL in men and < 50 mg/dL in women, or triglycerides ≥ 250 mg/dL, measured after at least ten hours of fasting, or previous diagnosis of dyslipidemia and taking lipid lowering agents. *Obesity* was having a body mass index (BMI) ≥ 30 kg/m^2^. The definition of *cigarette smoking* was ever having smoked more than 100 cigarettes. *Opium consumption* was defined as current or former use of opium through ingestion or smoking.

### Inclusion and exclusion

All patients in the THC coronary angiography databank enrolled from 2005 to 2015 who had an obstructive CAD—defined by an angiography report of more than 50% stenosis in at least one vessel—were included, except where the patient data were considered implausible. For this analysis, implausible values (age younger than 20 or older than 100, FBS less than 50 or higher than 600, LDL-C of less than 20 or higher than 500, HDL-C of less than 5 or greater than 100, total cholesterol of less than 50 or greater than 700, triglyceride level of less than 20 or greater than 1200, and creatinine level of less than 0.2 or greater than 10) were excluded from the study. This led to a total of 0.03% of the study population.

### Statistical analysis

Numbers and percentages were calculated and shown for categorical variables (sex, dyslipidemia, diabetes, hypertension, ever cigarette smoking, and ever opium use). Means and standard deviations (SD) were calculated and presented for continuous variables (age, BMI, total cholesterol, LDL-C, HDL-C, triglycerides, FBS). Mean of continuous variables and percentage of categorical variables were compared by sex using independent sample t-tests and chi-square tests, respectively.

To assess trends, numbers were tabulated by year, from 2005 to 2015. Linear regression was used to estimate the unadjusted, as well as age- and sex-adjusted annual change and 95% confidence interval (95% CI) in each variable by year. To show the trends for serum biomarkers (total cholesterol, LDL-C, HDL-C, triglycerides, and FBS), two-way graphs of the fitted values were drawn against the year. To assess the association of each predictor with age of CAD diagnosis, the study population was divided into three groups based on “age of CAD diagnosis” tertiles. Comparisons were made using one way ANOVA tests for continuous variables and chi-square tests for categorical variables. Further, linear regression was used to estimate the unadjusted and adjusted coefficients (95% CIs), with age being the outcome. For this latter analysis, continuous variables were standardized to have a mean (SD) of 0 (1). Given the collinearity of some variables (eg., LDL-C with total cholesterol), variance inflation factors (VIF) were calculated to exclude variables that increased VIF above 2. To better evaluate the association of ever opium use and the age of coronary artery disease diagnosis in subgroups, linear regression analysis was used and adjusted (for sex, BMI, LDL-C, HDL-C, triglycerides, dyslipidemia, FBS, diabetes mellitus, hypertension, and cigarette smoking, if applicable) coefficients (95% CI) were reported. *P*-value less than 0.05 or 95% CIs that did not include zero in linear regression were considered statistically significant. Analysis was done using Stata 16.1 College Station, Tx.

## Results

From January 2005 to December 2015, data for 90,408 unique patients who had at least 50% stenosis in one of the vessels were included in the THC coronary angiography databank. Of these, 314 (0.3%) were excluded because extreme outlier data, therefore 90,094 patients were included in this analysis. Table 1 shows the baseline characteristics for these patients. A total of 61,684 (68.5%) were men and 28,410 (31.5%) were women.

**Table 1:** Demographic and cardiovascular risk factor data for patients enrolled in Tehran Heart Center Databank from 2005 to 2015

	All Years	2005	2006	2007	2008	2009	2010	2011	2012	2013	2014	2015
All patients	90,094	7101	7621	8465	7838	8341	8482	9111	8674	8621	9301	6539
Age at diagnosis (n = 90,094, years)	61.0	60.5	60.1	60.6	60.3	61.0	61.6	61.9	61.2	61.5	61.4	61.1
	10.7	10.2	10.4	10.9	10.6	10.8	11.0	11.2	10.9	10.7	10.8	10.3
Sex (n = 90,094, % male)	68%	70%	70%	68%	69%	69%	67%	66%	68%	67%	68%	72%
Body mass index (n = 65,320, kg/m^2^)	27.8	27.3	27.2	27.5	27.5	27.7	27.8	28.0	28.0	28.1	28.1	27.9
	4.5	4.3	4.3	4.3	4.4	4.4	4.5	4.6	4.5	4.5	4.6	4.5
Total Cholesterol (n = 81,363, mg/dL)	174.3	197.3	191.2	186.0	179.5	173.7	171.4	169.6	168.4	164.0	166.0	162.3
	47.3	44.7	48.8	48.0	47.7	45.7	46.4	45.7	45.7	44.7	45.8	44.3
LDL Cholesterol (n = 79,458, mg/dL)	106.2	116.3	112.9	111.2	104.8	108.8	106.4	105.6	104.4	102.9	100.8	98.7
	38.7	37.7	41.1	39.5	40.9	39.2	37.9	38.6	37.9	37.0	37.0	36.3
HDL Cholesterol (n = 80,899, mg/dL)	40.1	42.4	42.0	41.5	41.4	40.6	40.1	39.6	39.0	38.7	38.3	39.2
	10.6	9.9	10.0	10.2	11.1	11.2	10.6	10.8	10.6	10.4	10.4	10.5
Triglycerides (n = 81,390, mg/dL)	162.8	201.0	186.0	170.2	173.2	161.7	161.2	156.0	151.1	150.4	151.7	147.3
	96.9	120.0	107.5	101.3	102.5	92.1	91.8	95.9	84.4	87.8	91.4	85.1
Dyslipidemia (n = 89,899, % yes)	64%	62%	69%	66%	64%	63%	60%	65%	69%	64%	64%	60%
Fasting blood sugar (n = 79,822, mg/dL)	122.0	122.8	121.4	122.3	123.7	121.7	121.1	121.8	122.1	120.9	122.8	122.3
	53.3	54.5	54.3	54.7	55.1	53.3	53.5	54.2	54.1	50.7	52.2	50.4
Diabetes mellitus (n = 89,977, % yes)	34%	31%	31%	31%	34%	33%	35%	36%	36%	36%	36%	36%
Hypertension (n = 90,001, % yes)	58%	53%	50%	52%	55%	57%	56%	63%	63%	62%	62%	58%
Ever cigarette smoking (n = 89,955, % yes)	38%	40%	40%	39%	38%	37%	38%	37%	38%	36%	36%	39%
Ever opium use (n = 87,348, % yes)	14%	14%	13%	14%	13%	14%	13%	13%	14%	14%	15%	16%

Numbers and percentages are shown for categorical variables (sex, dyslipidemia, diabetes, hypertension, ever cigarette smoking, and ever opium use). Means and standard deviations are presented for continuous variables (age; body mass index; total, LDL, HDL cholesterol; triglycerides; fasting blood sugar)

### Risk factor trends

#### Age at diagnosis

The mean (SD) of age at diagnosis for all years combined was 61.0 (10.7). Men were younger at diagnosis than in women, with a mean age of 60.1 in men and 63.2 in women (*p* < 0.001). While there were some slight fluctuations, mean age at diagnosis had an overall increasing trend during the study period. From 2005 to 2008, mean age at each year was below 61, while from 2009 to 2015, mean age was 61 or higher (Table 1). The sex-adjusted mean (95% CI) annual increase in age of CAD diagnosis was 0.12 (0.10, 0.14; *p* < 0.001), which was similar to unadjusted results (Table 2).

**Table 2:** Unadjusted and adjusted annual change in demographic and cardiovascular risk factors in patients enrolled in Tehran Heart Center Databank from 2005 to 2015

	Unadjusted annual change (95% CI)^a^	*P*-value	Adjusted annual change (95% CI)^b^	*P*-value
Age at diagnosis (n = 90,094, years)	0.12 (0.10, 0.15)	< 0.001	0.12 (0.10, 0.14)	< 0.001
Sex (n = 90,094, % male)	− 0.11 (− 0.21, − 0.01)	0.03	− 0.04 (− 0.14, 0.06)	0.43
Body mass index (n = 65,320, kg/m^2^)	0.08 (0.07, 0.10)	< 0.001	0.09 (0.08, 0.10)	< 0.001
Total Cholesterol (n = 81,363, mg/dL)	− 3.27 (− 3.38, − 3.17)	< 0.001	− 3.23 (− 3.33, − 3.13)	< 0.001
LDL Cholesterol (n = 79,458, mg/dL)	− 1.48 (− 1.56, − 1.39)	< 0.001	− 1.45 (− 1.54, − 1.37)	< 0.001
HDL Cholesterol (n = 80,899, mg/dL)	− 0.41 (− 0.44, − 0.39)	< 0.001	− 0.42 (− 0.44, − 0.40)	< 0.001
Triglycerides (n = 81,390, mg/dL)	− 4.51 (− 4.73, − 4.29)	< 0.001	− 4.35 (− 4.57, − 4.14)	< 0.001
Dyslipidemia (n = 89,899, % yes)	− 0.20 (− 0.31, − 0.10)	< 0.001	− 0.17 (− 0.27, − 0.07)	0.001
Fasting blood sugar (n = 79,822, mg/dL)	− 0.03 (− 0.15, 0.09)	0.65	− 0.02 (− 0.14, 0.10)	0.77
Diabetes mellitus (n = 89,977, % yes)	0.62 (0.51, 0.72)	< 0.001	0.58 (0.48, 0.68)	< 0.001
Hypertension (n = 90,001, % yes)	1.16 (1.06, 1.27)	< 0.001	1.05 (0.95, 1.15)	< 0.001
Ever cigarette smoking (n = 89,955, % yes)	− 0.29 (− 0.39, − 0.19)	< 0.001	− 0.16 (− 0.25, − 0.07)	0.001
Ever opium use (n = 87,348, % yes)	0.15 (0.08, 0.23)	< 0.001	0.23 (0.15, 0.30)	< 0.001

^a^All changes are annual. Changes for categorical variables (sex, dyslipidemia, diabetes, hypertension, ever cigarette smoking, and ever opium use) are percentages per year. Changes for continuous variables (age; body mass index; total, LDL, HDL cholesterol; triglycerides; fasting blood sugar) are using the unit shown in the first column

^b^Adjusted for age and sex

#### Sex

Overall, 68% of the patients were men and 32% were women. This percentage did not change appreciably over the course of the study (Table 1). The unadjusted and adjusted mean annual change in percentage of men were − 0.11% (*p* = 0.03) and − 0.04% (*p* = 0.43), respectively (Table 2).

#### Body mass index

The mean (SD) of BMI was 27.8 (4.5) kg/m^2^ for all years combined. Men had a lower mean BMI (27.0) than women (29.4) at enrollment (*p* < 0.001). There was an increasing trend for BMI, with mean BMI being close to 27.3 in 2005, but increasing to about 28.0 kg/m^2^ after 2011 (Table 1). The age- and sex-adjusted mean (95% CI) annual increase in BMI was 0.09 (0.08, 0.10; *p* < 0.001; Table 2).

#### Lipid profile and dyslipidemia

**Fig. 1:**
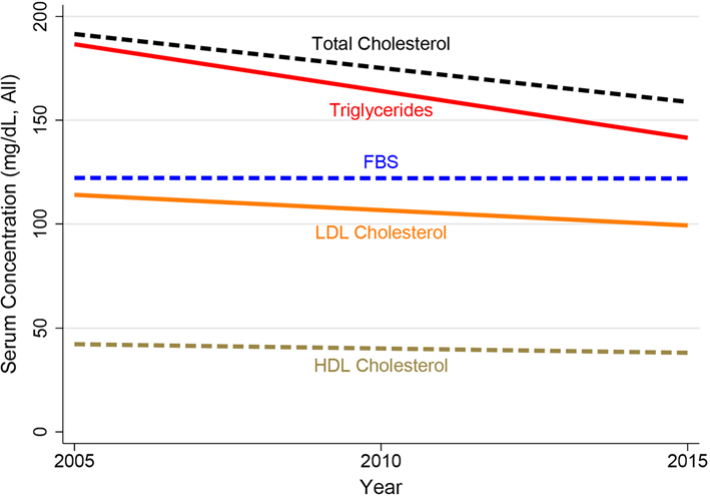
Overall trend of lipid profile and blood sugar in established coronary artery diseases patients, n = 90,094; 2005–2015

The mean (SD) of total cholesterol was 174.3 (47.3) mg/dL for all years. Men had a lower mean total cholesterol (169.5) than women (184.4) at enrollment (*p* < 0.001). Mean total cholesterol showed a clear decreasing trend over the study years, steadily declining from 197.3 mg/dL in 2005 to 162.3 mg/dL in 2015 (Table 1). The age- and sex-adjusted mean annual change in total cholesterol was − 3.23 (− 3.33, − 3.13; *p* < 0.001; Table 2 and Fig. 1).

The mean (SD) of LDL-C was 106.2 (38.7) mg/dL for all years. Men had a lower mean LDL-C (103.6) than women (111.7) at enrollment (*p* < 0.001). Mean LDL-C showed a decreasing trend over the study years, declining from 116.3 mg/dL in 2005 to 98.7 mg/dL in 2015 (Table 1). The age- and sex-adjusted mean (95% CI) annual change in LDL-C was − 1.45 (− 1.54, − 1.37; *p* < 0.001; Table 2 and Fig. 1).

**Fig. 2:**
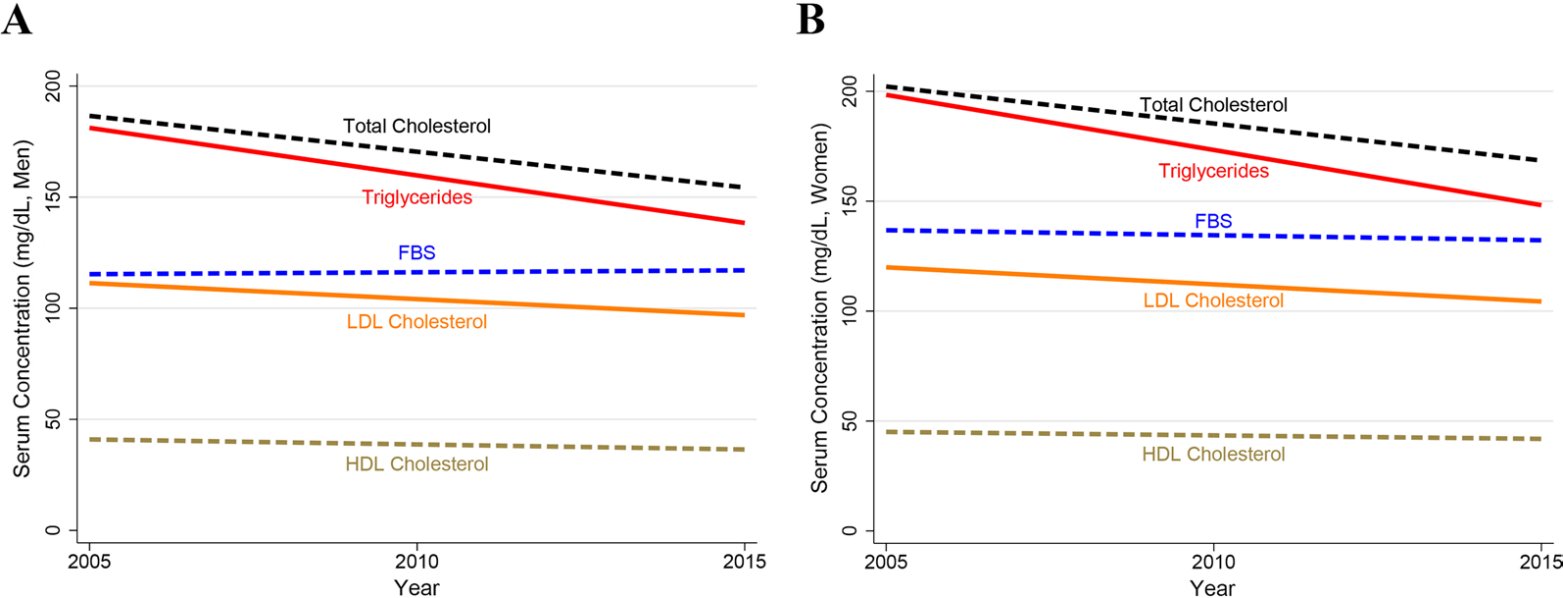
Trend of lipid profile and blood sugar in established coronary artery diseases patients; **A** Men (n = 61,684) vs. **B** Women (n = 28,410); 2005–2015

The mean (SD) of HDL-C was 40.1 (10.6) mg/dL for all years. Men had a lower mean HDL-C (38.6) than women (43.4) at enrollment (*p* < 0.001). Mean HDL-C showed an overall decreasing trend over the study years, with mean being 42.0 or higher in the first two years, while hovering around 39.0 in the last four years (Table 1). The age- and sex-adjusted mean (95% CI) annual change in HDL-C was − 0.42 (− 0.44, − 0.40; *p* < 0.001; Table 2 and Figs. 1 and 2).

The mean (SD) of triglycerides was 162.8 (96.9) mg/dL for all years. Men had a lower mean triglycerides level (158.6) than women (172.0) at enrollment (*p* < 0.001). Mean triglycerides had a clear and steep decreasing trend over the study years, steadily declining from 201.0 mg/dL in 2005 to 147.3 mg/dL in 2015 (Table 1). The age- and sex-adjusted mean (95% CI) annual change in triglycerides was − 4.35 (− 4.57, − 4.14); *p* < 0.001; Table 2 and Fig. 1).

Overall, 64% of the study population were classified as dyslipidemic, a diagnosis that included taking medications and previous history, in addition to current lipid profile. Men had lower prevalence of dyslipidemia (59.1%) than women (75.3%) at enrollment. A review of Table 1 does not show a clear increasing or decreasing pattern in the prevalence of dyslipidemia over the study years.

#### Blood sugar and diabetes

The mean (SD) of FBS was 122.0 (53.3) mg/dL and remained relatively constant over the years of the study. Men had a lower mean FBS (116.2) than women (134.4) at enrollment (*p* < 0.001). The mean annual FBS change was − 0.02 (− 0.14, 0.10; *p* = 0.77; Table 2 and Fig. 1 and 2). The overall prevalence of diabetes for all years was 34%. Men had a lower prevalence of diabetes (27.9%) than women (47.6%). This prevalence gradually increased from 31% in 2005 to 36% in 2015 (Table 1), with an annual average sex- and age-adjusted increase of 0.58% (0.48%, 0.68%; *p* < 0.001).

#### Hypertension

The overall prevalence of hypertension for all years was 58%. Men had a lower prevalence of hypertension (49.2%) than women (76.2%). The prevalence of hypertension gradually increased from low 50%s in the first three years of the study to around 60% in the last five years of the study (Table 1), with an annual average sex- and age-adjusted increase of 1.05% (0.95%, 1.15%; *p* < 0.001).

#### Ever cigarette smoking

The overall prevalence of a history of ever cigarette smoking was 38%. The prevalence of ever smoking was far higher in men (51.8%) than in women (7.6%). This prevalence gradually decreased from 40% in 2005 to 36% during the last few years of the study, except for 2015, which bounced back to 39% (Table 1). The average annual change in age- and sex-adjusted prevalence was − 0.16% (− 0.25%, − 0.07%; *p* < 0.001).

#### Opium use

The overall prevalence of a history of ever using opium was 14%. The prevalence of ever using opium was substantially higher in men (19.2%) than in women (2.0%). While there was no clear trend over the years (Table 1), the prevalence was higher toward the end of the study (16%) than in the beginning (14%). The average annual change in age- and sex-adjusted prevalence was 0.23% (0.15%, 0.30%; *p* < 0.001).

### Predictors of age of diagnosis

Table 3 shows the results of the prevalence of risk factors by age tertiles: ≤ 55 (n = 28,390), 56 to 65 (n = 30,343), and ≥ 66 (n = 31,361). The results show that younger patients are more likely to be men; have a higher BMI; have higher total cholesterol, higher LDL-C, higher triglycerides, and higher risk of dyslipidemia; and much more likely to be current smokers and opium users. In other words, younger patients had more risk factors, which is perhaps why they were diagnosed with CAD at earlier ages.

**Table 3:** Risk factors categorized by age tertiles

	Age tertiles					
	≤ 55 n = 28,390		56–65 n = 30,343		≥ 66 n = 31,361	
*Sex*						
Female	6424	(22.6%)	10,252	(33.8%)	11,734	(37.4%)
Male	21,966	(77.4%)	20,091	(66.2%)	19,627	(62.6%)
Body mass index (kg/m^2^)	28.1	(4.28)	27.9	(4.38)	27.2	(4.32)
Total cholesterol (mg/dL)	178.2	(48.37)	174.3	(47.11)	170.8	(46.20)
LDL cholesterol (mg/dL)	108.8	(39.78)	106.2	(38.47)	103.8	(37.73)
HDL cholesterol (mg/dL)	38.4	(10.01)	40.1	(10.51)	41.6	(11.04)
Triglyceride (mg/dL)	181.9	(110.49)	164.4	(94.79)	144.4	(81.36)
*Dyslipidemia*						
No	9453	(33.4%)	9942	(32.8%)	12,735	(40.7%)
Yes	18,880	(66.6%)	20,351	(67.2%)	18,538	(59.3%)
Fasting blood sugar (mg/dL)	119.4	(52.74)	125.8	(56.10)	120.6	(50.80)
*Diabetes Mellitus*						
No	20,299	(71.6%)	18,558	(61.2%)	20,416	(65.2%)
Yes	8064	(28.4%)	11,753	(38.8%)	10,887	(34.8%)
*Hypertension*						
No	15,353	(54.1%)	12,294	(40.6%)	10,455	(33.4%)
Yes	13,010	(45.9%)	18,022	(59.4%)	20,867	(66.6%)
*Ever cigarette smoking*						
No	14,097	(49.7%)	19,044	(62.8%)	22,767	(72.7%)
Current	9617	(33.9%)	6283	(20.7%)	3420	(10.9%)
Former	4641	(16.4%)	4974	(16.4%)	5112	(16.3%)
*Ever opium use*						
No	22,203	(80.7%)	25,215	(85.6%)	27,909	(91.8%)
Current	4148	(15.1%)	3473	(11.8%)	2128	(7.0%)
Former	1152	(4.2%)	766	(2.6%)	354	(1.2%)

Numbers and percentages are shown for categorical variables (sex, dyslipidemia, diabetes, hypertension, ever cigarette smoking, and ever opium use). Means and standard deviations are presented for continuous variables (age; body mass index; total, LDL, HDL cholesterol; triglycerides; fasting blood sugar)

All *p*-values were < 0.001 in Chi square and one-way ANOVA analysis

Table 4 shows the predictors of age of diagnosis. Men were diagnosed with CAD approximately 3 years younger than women with a mean difference (95% CI) of − 3.12 (− 3.27, − 2.97). After adjusting for other variables in the model, being a male was still associated with earlier age at diagnosis, but only less than one year younger, with a mean difference (95% CI) of − 0.89 (− 1.09, − 0.68).

**Table 4:** Unadjusted and adjusted predictors of age of diagnosis with coronary artery disease at Tehran Heart Center from 2005 to 2015

	Unit	Unadjusted (95% CI)^a^	*P*-value	Adjusted (95% CI)^b^	*P*-value
Sex (n = 90,094)	Male (vs. female)	− 3.12 (− 3.27, − 2.97)	< 0.001	− 0.89 (− 1.09, − 0.68)	< 0.001
Body mass index (n = 65,320)	One SD (4.5 kg/m^2^)	− 1.07 (− 1.15, − 0.99)	< 0.001	− 1.52 (− 1.60, − 1.43)	< 0.001
Total Cholesterol (n = 81,363)	One SD (47.3 mg/dL)	− 0.78 (− 0.85, − 0.71)	< 0.001	Excluded^c^	–
LDL Cholesterol (n = 79,458)	One SD (38.7 mg/dL)	− 0.64 (− 0.72, − 0.57)	< 0.001	− 0.43 (− 0.52, − 0.35)	< 0.001
HDL Cholesterol (n = 80,899)	One SD (10.6 mg/dL)	1.39 (1.32, 1.47)	< 0.001	0.71 (0.62, 0.79)	< 0.001
Triglycerides (n = 81,390)	One SD (96.9 mg/dL)	− 1.87 (− 1.94, − 1.80)	< 0.001	− 1.35 (− 1.45, − 1.26)	< 0.001
Dyslipidemia (n = 89,899)	Yes (vs. No)	− 1.65 (− 1.80, − 1.50)	< 0.001	− 0.81 (− 1.00, − 0.62)	< 0.001
Fasting blood sugar (n = 79,822)	One SD (53.3 mg/dL)	0.12 (0.05, 0.19)	0.002	− 0.32 (− 0.42, − 0.22)	< 0.001
Diabetes mellitus (n = 89,977)	Yes (vs. No)	1.24 (1.10, 1.39)	< 0.001	1.05 (0.84, 1.26)	< 0.001
Hypertension (n = 90,001)	Yes (vs. No)	3.98 (3.84, 4.12)	< 0.001	3.54 (3.37, 3.71)	< 0.001
Ever cigarette smoking (n = 89,955)	Yes (vs. No)	− 4.41 (− 4.55, − 4.26)	< 0.001	− 2.76 (− 2.95, − 2.57)	< 0.001
Ever opium use (n = 87,348)	Yes (vs. No)	− 4.22 (− 4.43, − 4.02)	< 0.001	− 2.20 (− 2.44, − 1.96)	< 0.001

^a^All differences are shown for age at diagnosis by unit in each predictor

^b^Mutually adjusted for all other variables

^c^Total cholesterol was not included in the model due to high collinearity with other variables

In the adjusted model, one SD increase in BMI (4.5 kg/m^2^) was associated with approximately 1.5 years of earlier at diagnosis, with a 95% (CI) of − 1.52 (− 1.60, − 1.43). Other factors associated with younger age at diagnosis and their estimated coefficients (95% CIs) were: serum LDL-C (each SD, 38.7 mg/dL) with − 0.43 (− 0.52, − 0.35) years; serum triglycerides (96.9 mg/dL) with − 1.35 (− 1.45, − 1.26) years; dyslipidemia (yes vs. no) with − 0.81(− 1.00, − 0.62) years; FBS (53.3 mg/dL) with − 0.32 (− 0.42, − 0.22) years; ever cigarette smoking with − 2.76 (− 2.95, − 2.57) years; and ever opium use with − 2.20 (− 2.44, − 1.96) years.

Factors associated with an older age at diagnosis in the adjusted models and their respective coefficients (95% CI) were higher HDL-C (10.6) with 0.71 (0.62, 0.79) years; diabetes mellitus with 1.05 (0.84, 1.26), and hypertension with 3.54 (3.37, 3.71).

To further investigate the effect of opium use, we evaluated the association in various subgroups (Table 5). It was associated with earlier age of CAD diagnosis in nearly all subgroups, except for those who were 55 or younger.

**Table 5:** Association of ever opium use and the age of coronary artery disease diagnosis at Tehran Heart Center: subgroup analyses

	Adjusted coefficient (95% CI)^a^	*p*-value
*Age, tertiles (years)*		
≤ 55	0.18 (− 0.02, 0.38)	0.075
56–65	− 0.17 (− 0.29, − 0.05)	0.004
≥ 66	− 1.07 (− 1.31, − 0.83)	< 0.001
*Sex*		
Male	− 2.23 (− 2.48, − 1.98)	< 0.001
Female	− 1.14 (− 2.11, − 0.16)	0.022
*Dyslipidemia*		
Yes	− 1.98 (− 2.27, − 1.68)	< 0.001
No	− 2.57 (− 2.97, − 2.17)	< 0.001
*Diabetes mellitus*		
Yes	− 1.53 (− 1.96, − 1.11)	< 0.001
No	− 2.41 (− 2.70, − 2.12)	< 0.001
*Hypertension*		
Yes	− 2.10 (− 2.43, − 1.77)	< 0.001
No	− 2.26 (− 2.60, − 1.91)	< 0.001
*Cigarette smoking*		
Ever	− 2.44 (− 2.71, − 2.17)	< 0.001
Never	− 1.13 (− 1.63, − 0.63)	< 0.001

^a^Using linear regression models, adjusted for sex, body-mass index, low-density lipoprotein cholesterol, high-density lipoprotein cholesterol, triglycerides, dyslipidemia, fasting blood sugar, diabetes mellitus, hypertension, and cigarette smoking, if applicable

## Discussion

This study is the largest hospital-based serial cross-sectional study of patients with established CAD thus far conducted in Iran. Despite its limitations, the study results give us unique insights about comparing the prevalence in CAD patients with the general population, comparison of risk factors among men and women, trends of risk factors among CAD patients, the impact of neglected risk factors such as opium, and the impact of these risk factors on age of CAD diagnosis.

The associations of traditional risk factors with cardiovascular disease in Iran are well-established [10–15]. In 2005, hypertension was responsible for 80,000, hypercholesterolemia for 34,000, diabetes for 34,000, and smoking for 11,000 deaths in Iran in 2005 [11]. Nationwide, the risk of cardiovascular disease attributed to hypertension, high LDL-C, diabetes, and smoking were estimated as 36%, 24.1%, 9.9%, and 5.5%, respectively [10].

### Comparisons with general population

The prevalence of CAD risk factors have been studied in cross-sectional samples of the Iranian general population, such as the STEPS studies [16]. As expected, the prevalence of CAD risk factors was higher in CAD patients of our study compared to the general population; for example, the prevalence rates of hypertension, dyslipidemia, cigarette smoking, and diabetes mellitus were, respectively, 58%, 64%, 38%, and 34% in our study, compared with 17%, 33%, 12%, and 10% in general population; and 55%, 48%, 16%, and 21% in older adults (55–65 years), according to STEPS study 2007 [16]. In 2016, a systematic review and pooled analysis reported the national age-standardized hypertension prevalence of 24% and 29%, among Iranian adults (> 25 years) in men and women, respectively [17]. Another national study reported the hypertension prevalence of 23% and 50% in 30–55 and > 55 year-old populations, respectively [18]. A systematic review demonstrated that the estimated national prevalence and 95% confidence intervals for hypercholesterolemia (≥ 200 mg/dL), hypertriglyceridemia (≥ 150 mg/dL), high levels of LDL-C (≥ 130 mg/dL) and low levels of HDL-C (< 40 mg/dL in male and < 50 mg/dL in female) in Iranian people are 41.6% (36.1–47.0), 46.0% (43.3–48.7), 35.5% (24.0–47.1) and 43.9% (33.4–54.4), respectively, among ≥ 15 year-old population [19]. According to the International Diabetes Federation 2019 report, diabetes national prevalence in Iranian adults 20–79 years was 9.4% (7.4%-12.3%) and more than 5 million Iranian people had diabetes. The Middle East and North Africa region has the highest age-adjusted diabetes prevalence worldwide and the number of people with diabetes in this region, by 2045, will increase by 96.5%. The prevalence of diabetes among > 50-year-old population in this region is almost 25% [20].

### Comparing men and women with CAD

Being a man is an established risk factor for CAD. In our study, 68% of all patients were men, whereas approximately 50% of the general population are men. Men were diagnosed with CAD approximately three years earlier than women. Even after adjustment for a host of risk factors, being a man was still associated with a one-year earlier diagnosis. This may reflect either incomplete adjustment for risk factors, or an inherited tendency for higher risk of CAD in men.

As expected, the prevalence of ever tobacco smoking (51.8% vs. 7.6%) and ever opium consumption (19.2% vs. 2.0%) was substantially higher in men than in women. These numbers are consistent with findings from population-based studies. In a meta-analysis published in 2013, the prevalence of cigarette smoking was reported to be 19.8% to 21.7% in men and 0.94% to 3.6% in women [21]. A recent systematic analysis from the Global Burden of Disease Study reported that the prevalence rates of smoking in Iran were 5% and 25% in females and males aged 15 years or older, respectively. Almost 1.6 million women and 8.7 million men are current smokers in the country [22]. This gender difference is primarily because consuming opium and smoking tobacco was a more acceptable behavior for men than for women. These two risk factors explain a substantial amount of the difference in age of diagnosis between men and women.

Unlike the global prevalence of diabetes in men and women (9.6% vs. 9.0%, respectively) [23], the prevalence of diabetes in Iranian women is 1.7% higher than men [24] mainly because a greater obesity rate among Iranian women [25]. Therefore, our results about the higher BMI and diabetes prevalence in women are in line with other national studies [16, 24–27].

Other risk factors, however, were more common in women than in men in our study. For example, compared to women, men had a lower prevalence of dyslipidemia (59.1% vs. 75.3%) and hypertension (49.2% vs. 76.2%). These differences do not reflect differences in the general population. For example, the prevalence of diabetes mellitus in the general population is higher in men than in women [12, 13]. Rather, observed pattern is because our study participants all had CAD. Women who were less likely to be cigarette smokers and opium users had other risk factors to expose them to CAD. In recent reports from China National Stroke Screening and prevention project (CNSSPP) in 2019, similar to our results, prevalence of dyslipidemia was higher among woman than men (54% vs. 46% in urban and 52% vs. 48% in rural area) [28]. Likewise, in a study from neighboring Azerbaijan, Zeynalov and colleagues reported that the prevalence of hypertension among CAD population was significantly higher in women than men (66.2% vs. 58.4%, *p* < 0.05) [29].

### The trends of risk factors over the study period

Over the 11-year study period, the average serum concentration of total cholesterol, LDL-C, HDL-C, and triglycerides declined substantially. These trends are similar to those found in population-based serial cross-sectional studies in Iran. For example, in the STEPS (2007–2016) studies in Iran, total cholesterol, LDL-C, and triglycerides decreased substantially over the course of the study [30]. Decreasing trend of non-HDL-C was also observed in most western countries, Japan, and South Korea [31]. This decrease could be mainly attributed to statin prescription and dietary modifications, e.g., replacement of saturated with unsaturated fats and reduction in trans-fats. In a recent study on non-communicable disease in Iran, approximately half (46.5%) of 40–70-year Iranians were eligible for moderate- to high-intensity statin therapy based on American College of Cardiology and the American Heart Association (ACC/AHA) guideline [32]. Serum HDL-C levels, however, changed only modestly. HDL-C has mostly genetic determinants but lower physical activity, smoking, and carbohydrates consumption may also lower HDL-C levels [30, 31].

The prevalence of several other risk factors, i.e., higher BMI, diabetes, and hypertension was increasing in our study population. While studies conducted in the general population also show increasing trends of BMI and FBS, trends of hypertension have been on the decline. Data from the STEPS studies in the Iranian general population [30] show that, from 2007 to 2016, BMI was increasing in non-diabetic men population; but there was no significant trend in non-diabetic women, nor in the diabetic population. In this study, FBS had an increasing trend among non-diabetic population [30]. However, both systolic and diastolic pressure in the general population were decreasing [33]. In another report by Esteghamati et al. in 2016, in adult population of Iran the prevalence of hypertension gradually decreased from 25.7% in 2005 to 24.1% in 2011 [34]. The results of our study are different from the general population, perhaps because given that lipid risk factors are declining, other risk factors need to be present for CAD to happen, so we may see a higher prevalence of hypertension in our patients. Another reason for this difference may be that we do not have the actual measures blood pressures in our database; only hypertension (yes vs. no) has been registered in a dichotomous fashion.

Based on the STEPS studies, there was a significant reduction in cigarette smoking prevalence in the Iranian adults (25–65 years), for example, in non-diabetic men, from 29.1% in 2007 to 21.8% in 2016 [30]. Our numbers are slightly different, however, it has to be noted that our exposure in this study was “ever use of cigarettes”, which is not expected to change much and is higher than “current smoking” prevalence. Likewise, given that our questions were asking about “ever opium use”, we did not expect a major trend over time.

### Risk factors and age of diagnosis

One would expect that the presence of risk factors will be associated with a lower age of CAD diagnosis. Assuming that the date of entry into the THC database is the age of diagnosis, we observed that most known risk factors—such as male sex, total cholesterol, LDL-C, triglycerides, dyslipidemia, FBS, tobacco smoking, and opium consumption—were all associated with lower age at diagnosis.

Of particular interest was opium use, which after adjustment for other risk factors, was associated with 2.2 years of reduction in age of diagnosis. Opium consumption is very common in Iran [35], and has recently been shown to be a risk factor of cardiovascular diseases, independent to traditional risk factors [36]. Most people, and even some physicians, are unaware of this finding. Opium has been traditionally used to alleviate pain resulting from chronic diseases, including chest pain. Therefore, some people may mistakenly believe that opium “cures” their disease, while it is only a pain reliever and indeed is a cause of their CAD [37].

Surprisingly, the presence of diabetes mellitus and hypertension were associated with later age of onset of CAD. Given that higher FBS was associated with a younger age of CAD onset, the observation with diabetes mellitus may be explained by the fact that those diagnosed as diabetics might have received treatment earlier. Therefore, it may be the treatment that is associated with later age of onset. Unfortunately, we did not have recorded blood pressure in our database; we only had hypertension coded in a binary fashion (yes vs. no). Therefore, it is difficult to make a judgment about hypertension. However, this association, like that of diabetes, might also be explained by earlier diagnosis and treatment.

### The overall trends in age of diagnosis

Some risk factors (e.g., lipid profile) were moving in the right direction, while others (e.g., BMI) were moving in the wrong direction over the course of this study. Overall, the age of diagnosis of CAD slightly increased over the course of this study, a finding to celebrate. However, changes in age of diagnosis during this period may reflect changes in risk factors over the preceding three or four decades.

### Strengths and limitations of the study

To our knowledge, this is the first large-scale study that has enrolled patients with established CAD in Iran. The large number of patients, documentation of CAD with angiography which is the gold standard for evaluation of atherosclerotic CAD, and data registry in a specialized “Risk Factors Clinic” over 11 years, are among the strengths of this study. THC is a referral educational heart hospital in Tehran, and the patients are referred to this center are from all parts of the country, hence the results of the study may be generalized to Iranian population with documented CAD.

This study has some limitation too. The absolute value of blood pressure (systolic and diastolic) was not available for all patients. This study was a serial cross-sectional study, and thus did not capture development of hypertension or diabetes after the initial visit. Another important issue is lack of data about duration and quantity of opium use and cigarette smoking, which both are probably related to CAD occurrence. We did not have accurate data to report on alcohol consumption. However, a very small percentage of the population may have appreciable consumption of alcohol, as the majority of the population are Muslims who do not drink alcohol, and alcohol is not readily available due to legal restrictions.

### Conclusions and recommendations

Women are diagnosed with CAD at older ages than men, and had several more risk factors at the time of CAD diagnosis. In other words, being a female was protective against CAD, and CAD could happen in women only when a few risk factors came together. We observed a substantial decreasing trend of total cholesterol, LDL-C, and triglycerides over the course of the study, perhaps contributing to a later onset of the disease over time. Perhaps the most important finding of this study is confirming opium use as a risk factor for earlier age of onset of CAD. This is a relatively recent finding, and most people and even some physicians are unaware of it. Recommendations for lowering the use of opium should be considered along other measures to control CAD, such as taking statins, taking blood pressure and diabetes medications in those who need it, as well as reduction in smoking and enhancing exercise. Considering the use of “polypill”, a fixed-dose combination of an anti-platelet, antihypertensives, and lipid-lowering drugs may also be considered, particularly in rural populations with low access to physicians and low compliance.

## Data Availability

All data associated with the article is available in Excel and SPSS format if required.

## Abbreviations

CAD: Coronary artery disease
LMIC: Low- to middle-income countries
YLD: Years lived with disability
THC: Tehran Heart Center
BMI: Body-mass index
LDL-C: Low-density lipoprotein cholesterol
HDL-C: High-density lipoprotein cholesterol
FBS: Fasting blood sugar
HbA1C: Glycated hemoglobin A1C
SD: Standard deviations
CI: Confidence interval
VIF: Variance inflation factors
CNSSPP: China National Stroke Screening and prevention project
ACC/AHA: American College of Cardiology and the American Heart Association
